# Effects of endurance training under calorie restriction on energy substrate metabolism in mouse skeletal muscle and liver

**DOI:** 10.1186/s12576-024-00924-5

**Published:** 2024-06-07

**Authors:** Kenya Takahashi, Yu Kitaoka, Hideo Hatta

**Affiliations:** 1 Department of Sports Sciences, Graduate School of Arts and Sciences, The University of Tokyo, 3-8-1, Komaba, Meguro-ku, Tokyo, 153-8902 Japan; 2 Department of Human Sciences, Kanagawa University, 3-27-1, Rokkakubashi, Kanagawa-ku, Yokohama, Kanagawa 221-8686 Japan

**Keywords:** Endurance training, Calorie restriction, Mitochondria, Enzyme, Transporter, Gluconeogenesis, Skeletal muscle, Liver

## Abstract

We investigated whether calorie restriction (CR) enhances metabolic adaptations to endurance training (ET). Ten-week-old male Institute of Cancer Research (ICR) mice were fed ad libitum or subjected to 30% CR. The mice were subdivided into sedentary and ET groups. The ET group performed treadmill running (20–25 m/min, 30 min, 5 days/week) for 5 weeks. We found that CR decreased glycolytic enzyme activity and monocarboxylate transporter (MCT) 4 protein content, while enhancing glucose transporter 4 protein content in the plantaris and soleus muscles. Although ET and CR individually increased citrate synthase activity in the plantaris muscle, the ET-induced increase in respiratory chain complex I protein content was counteracted by CR. In the soleus muscle, mitochondrial enzyme activity and protein levels were increased by ET, but decreased by CR. It has been suggested that CR partially interferes with skeletal muscle adaptation to ET.

## Background

Weight loss is beneficial for preventing metabolic disorders in overweight and obese individuals [1, 2] and improving the power-to-body weight ratio and energy cost for a given workload in athletes [3, 4]. Calorie restriction (CR) is an effective means of inducing a negative energy balance, resulting in a decline in body and fat weight [5]. Additionally, CR exerts profound effects on energy metabolism. Studies have shown that CR results in increased mitochondrial content in several rodent tissues [6, 7] and can upregulate the expression of genes encoding proteins implicated in mitochondrial function in human skeletal muscle [8, 9]. Increasing energy expenditure through endurance training (ET) is another strategy to achieve a negative energy balance. ET not only reduces body and fat weight [1, 10, 11] but also enhances muscle metabolic capacity. Numerous studies have shown that ET improves mitochondrial function and metabolite transport in skeletal muscle [12–14]. In practical settings, ET and CR are used concurrently in the athletic and general populations. However, the relation between the ET and CR is not well understood. Therefore, the aim of this study was to investigate the effects of ET on metabolic adaptations in the skeletal muscle and liver during CR.

In the current study, we first determined the feeding patterns of mice that underwent CR. We then assessed diurnal changes in plasma substrates and energy deposits (glycogen and triglycerides) in muscle and liver. Next, we performed respiratory gas analysis in mice undergoing 5 weeks of CR and ET to evaluate substrate metabolism. To further understand the mechanisms by which CR and/or ET induce metabolic adaptations, we evaluated enzyme activities together with metabolite transport proteins in the skeletal muscle. For these analyses, we used both glycolytic and oxidative muscles, because their metabolic characteristics may cause different responses to CR and ET. Given that the liver is capable of converting substrates into other forms depending on nutritional status, we measured proteins involved in gluconeogenesis and fatty acid synthesis in the hepatic tissue.

## Methods

### Animals

All the experiments were approved by the Animal Experimental Committee of the University of Tokyo (No. 2021-1). Mice used in this study were male Institute of Cancer Research (ICR) mice (10 weeks old) bred in an animal facility at the University of Tokyo. Individual animals were housed under a 12:12 h light/dark cycle (dark: 7:00 AM to 7:00 PM) in an air-conditioned room (23 °C). Prior to the experiments, all the animals were allowed ad libitum (AL) access to a standard chow diet (MF; Oriental Yeast, Tokyo, Japan) and water.

### Experimental protocol

#### Experiment 1: Feeding pattern and substrate concentration

**Fig. 1:**
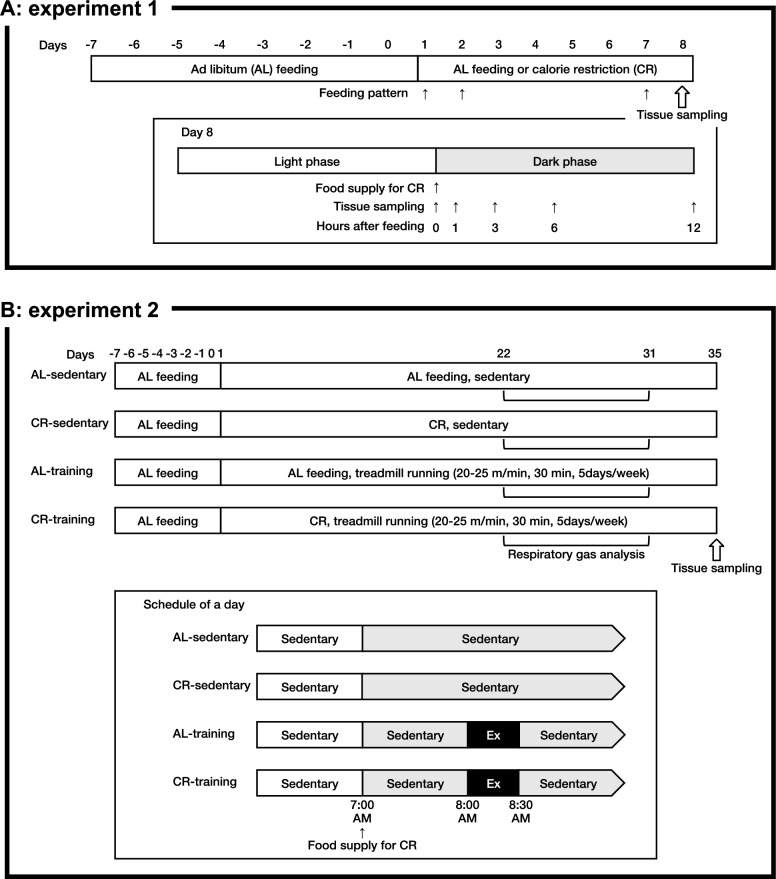
Schematic overview of the experiments. Schematic outlines of Experiment 1 (**A**) and Experiment 2 (**B**)

The experimental procedure is illustrated in Fig. 1A. Previous reports have shown that rodents provide unlimited access to food consumed over the entire day, whilst those undergoing CR typically eat their entire daily diet within several hours of feeding [15, 16]. Therefore, in this study, we first determined the feeding patterns and diurnal changes in substrate levels in the circulation and tissues. The animals were divided into the AL (n = 30) or CR (n = 30) group. Daily AL food consumption of all animals was measured over the one-week acclimation period. From days 1 to 8, at the onset of the dark phase (7:00 AM), each CR group animal provided 70% of the average daily food consumption in the acclimation period. AL group animals had free access to food throughout the experimental period. On days 1, 2, and 7, the food consumption of the 10 animals in each group was measured at 1, 3, 6, and 12 h after feeding. On day 8, before (0 h) and after (1, 3, 6, and 12 h) the daily food supply in the CR group, the animals were anesthetized by isoflurane inhalation and euthanized by blood removal from the inferior vena cava. The blood was then centrifuged at 3000×*g* for 20 min at 4 °C to obtain plasma samples. The tissues were harvested, rapidly frozen in liquid nitrogen, and then stored at − 80 °C until analysis.

#### Experiment 2: Training experiment under calorie restriction

Figure 1B illustrates a schematic diagram of the experiment. The animals were assigned to one of the following four groups: AL-sedentary (AL-Sed; n = 10), CR-sedentary (CR-Sed; n = 11), AL-training (AL-Tr; n = 10), or CR training (CR-Tr; n = 9). Daily AL food intake was measured during the one-week acclimation period. From day 1 of the experiment, at the onset of the dark phase (7:00 AM), CR group animals were provided with 70% of their average daily food consumption in the acclimation period. To prevent low energy availability during exercise (so-called “train-low”), the animals in the training groups ran on a treadmill (MK-680; Muromachi Kikai Co., Inc., Tokyo, Japan) 1 h after the onset of providing the daily food allotment to the CR mice. Treadmill running was performed for 30 min, five days per week for 5 weeks, at a speed of 20 m/min in the first training week and 25 m/min in the remaining training sessions. The training protocol was based on a previous study [17]. For ICR mice, running speed at maximal oxygen uptake and critical speed are 31.7 m/min and 24.1 ± 4.6 m/min, respectively [18, 19]. Thus, the present exercise protocol was most likely completed by all the mice in the training groups. Respiratory gas at rest was analyzed from days 22 to 31 as described below. Twenty-two hours after the last exercise session (before providing daily food to the CR group), the animals were anesthetized by isoflurane inhalation and euthanized by blood removal from the inferior vena cava. The blood was then centrifuged at 3000×*g* for 20 min at 4 °C to obtain plasma samples. The tissues were harvested, weighed on a scale, rapidly frozen in liquid nitrogen, and then stored at − 80 °C until analysis.

### Respiratory gas analysis

From the onset of the dark phase (7:00 AM), the animals were placed individually in an air-sealed metabolic chamber (AC-001M; Muromachi Kikai, Tokyo, Japan) for 24 h (dark–light: 12–12 h). Inspired O_2_ and expired CO_2_ levels were recorded every 5 min using a metabolism-measuring system (MK-5000RQ; Muromachi Kikai) at an airflow rate of 0.5 L/min. The respiratory exchange ratio (RER) was calculated using the expired CO_2_/inspired O_2_ (VCO_2_/VO_2_). Carbohydrate and lipid oxidation, as well as energy expenditure, were calculated as previously described [20], with minor modifications. Carbohydrate oxidation (kcal/h) = [4.55 VCO_2_ (ml/min) − 3.21 VO_2_ (ml/min)] × 3.74 (kcal/g) × 60 (min); lipid oxidation (kcal/h) = [1.67 VO_2_ (ml/min) − VCO_2_ (ml/min)] × 9.5 (kcal/g) × 60 (min); energy expenditure (kcal/h) = [3.91 VO_2_ (ml/min) + 1.10VCO_2_ (ml/min)] × 60 (min).

### Determination of plasma substrates

The plasma glucose level was assessed using a glucose CII kit (Fujifilm Wako, Osaka, Japan). Plasma free fatty acid (FFA) and triglyceride levels were quantified using the NEFA c-test (Fujifilm Wako) and LabAssay Triglyceride kit (Fujifilm Wako), respectively.

### Muscle and liver glycogen

Tissue glycogen content was quantified as previously described [21]. The whole gastrocnemius muscle or a liver fragment (approximately 30 µg) was dissolved in 30% (w/v) KOH solution saturated with Na_2_SO_4_. The solution was heated at 100 °C for 10 min. After the addition of 99% (v/v) ethanol, it was placed on ice for 30 min, followed by spinning at 10,000×*g* (10,379 rpm, r = 83 mm) for 10 min at 4 °C. After removal of the supernatant, the pellet was dissolved in 1 N HCl and heated at 100 °C for 2 h to hydrolyze glycogen to glucose. The solution was then neutralized with 1 N NaOH, and the glucose concentration was evaluated with a glucose CII kit (Fujifilm Wako).

### Muscle and liver triglyceride

Tissue triglyceride concentrations were analyzed as previously described [22]. Whole gastrocnemius muscle or a liver fragment (approximately 30 µg) was homogenized in a buffer containing 5% (v/v) NP-40 substitute (145-09701; Fujifilm Wako) using a µT-01 bead crusher (TAITEC, Saitama, Japan). The homogenate was heated (100 °C for 5 min) and cooled (room temperature: 25 °C) twice to solubilize the lipids. Following centrifugation at 10,000×*g* (10,379 rpm, r = 83 mm) for 2 min, the supernatant triglycerides were assayed using a kit (LabAssay™ Triglyceride Kit, Fujifilm, Wako).

### Enzyme activity assays

Whole plantaris and soleus muscles were homogenized in 100 times (vol/wt) of 100 mM phosphate buffer (pH 7.6) using bead crusher (TAITEC). The homogenates were freeze-thawed twice using liquid nitrogen and were centrifuged at 1000×*g* (3282 rpm, r = 83 mm) for 10 min at 4 °C. The supernatant was recovered and used for enzyme assays. The maximal activities of hexokinase, phosphofructokinase, lactate dehydrogenase, citrate synthase, cytochrome c oxidase, β-hydroxyacyl-CoA dehydrogenase, and total carnitine palmitoyltransferase were measured as previously described [23].

### Western blotting

Whole plantaris and soleus muscles, as well as liver fragments (approximately 100 mg), were homogenized in 20 volumes (vol/wt) of ice-cold RIPA lysis buffer (#20-188; Merck Millipore, Tokyo, Japan) supplemented with a protease inhibitor mixture (cOmplete Mini, ETDA-free; Roche Applied Science, Indianapolis, IN, USA). The homogenates were continuously mixed on ice by rotation for 60 min. Following the centrifugation at 1500×*g* (4020 rpm, r = 83 mm) at 4 °C for 20 min, the total protein concentration of the supernatant was measured using a BCA protein assay kit (TaKaRa BIO Inc., Shiga, Japan). The same amount of protein was loaded onto a sodium dodecyl sulfate–polyacrylamide gel and electrophoretically separated. Proteins were transferred onto polyvinylidene difluoride membranes and western blotting was carried out using a standard procedure as previously outlined [24]. The antibodies used in the present study are as follows: The blots were scanned and quantified using ChemiDoc XRS (Bio-Rad Laboratories, Hercules, CA, USA) and Quantity One® software (version 4.5.2; Bio-Rad Laboratories). The stain-free gels were visualized to verify consistent loading.

### Primary and secondary antibodies

Western blotting was performed using primary antibodies against peroxisome proliferator-activated receptor γ coactivator 1-α (PGC-1α; #516557) and glucose transporter 4 (GLUT4; #07-1404) from Merck Millipore, total OXPHOS Rodent WB Antibody Cocktail (#ab110413) from Abcam (Cambridge, UK), fatty acid translocase/cluster of differentiation 36 (FAT/CD36; #66395-1-Ig), glucose 6-phosphatase (G6Pase; #22169-1-AP), phosphoenolpyruvate carboxykinase cytosolic form (PEPCK-C; #16754-1-AP), phosphoenolpyruvate carboxykinase mitochondrial form (PEPCK-M; #14892-1-AP), pyruvate carboxylase (PC; #16588-1-AP), fatty acid synthase (FAS; #10624-2-AP), ATP citrate lyase (ACLY; #15421-1-AP) from Proteintech Japan, fructose-1,6-bisphosphatase (FBPase; #sc-166097) from Santa Cruz Biotechnology (Santa Cruz, CA, USA) and acetyl-CoA carboxylase (ACC; #3662) from Cell Signaling Technology Japan. Primary antibodies against monocarboxylate transporter (MCT)-1 and MCT-4 were raised in rabbits against the C-terminal regions of the respective MCTs (Qiagen, Tokyo, Japan) and were used in our previous studies [21, 25–27]. Rabbit anti-goat IgG (H&L) (#A102PT; American Qualex, San Clemente, CA, USA) and mouse anti-goat IgG (H&L) (#A106PU; American Qualex) were used as secondary antibodies.

### Statistical analysis

All data are presented as means ± the standard deviation (SD) of the mean. A two-way analysis of variance (ANOVA) was used to determine the interaction and main effects of time and treatment. When an interaction was found to be significant, Bonferroni’s multiple comparison test was performed to identify differences between groups. To compare the training experiments, a two-way ANOVA (CR × ET) was performed, followed by the Tukey–Kramer multiple comparison test. Comparisons between two groups were made using the Student’s *t*-test. All statistical analyses and figure visualizations were performed using GraphPad Prism software (Ver. 10.1.0, Macintosh; GraphPad Software, La Jolla, CA, USA). Statistical significance was set at p < 0.05.

## Results

### Experiment 1

#### Feeding pattern

**Fig. 2:**
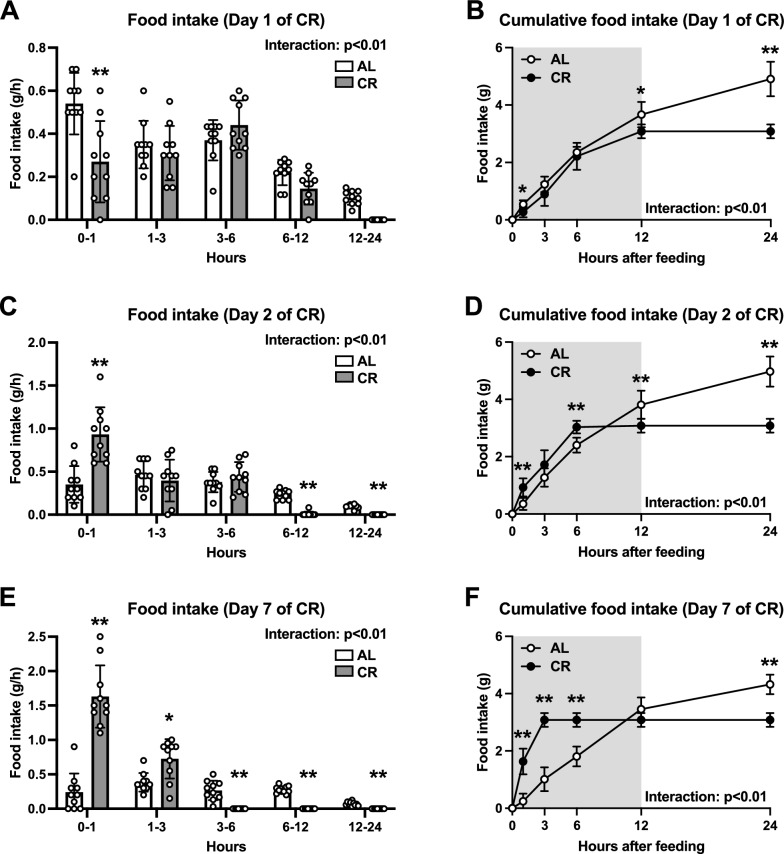
Feeding pattern in Experiment 1. Food intake (**A**, **C**, **E**) and cumulative food intake (**B**, **D**, **F**) on days one (**A**, **B**), two (**C**, **D**), and seven (**E**, **F**). Data are expressed as means ± the standard deviation (SD) (n = 10). Two-way analysis of variance (ANOVA) was performed to detect differences between two groups at the same time point. **p < 0.01, *p < 0.05: significant difference between the two groups within the same time point

To better understand the animal characteristics, we first assessed the feeding pattern during the seven days of calorie restriction. On day 1 of the CR, the animals in the CR group consumed almost all the daily food allotments within 12 h of feeding, whereas those in the AL group continued to consume chow throughout the day (Fig. 2A and B). On day 2 of CR, the animals in the AL group exhibited a feeding pattern similar to that observed on day 1, whereas the feeding pattern of the animals in the CR group shifted toward an earlier time point after feeding (Fig. 2C and D). Similarly, on day 7, the animals in the CR group ate all the daily food allotments within 3 h, whereas the feeding patterns of the animals in the AL group remained unchanged (Fig. 2E and F). Overall, the CR shifted the feeding pattern to an earlier time after the onset of feeding.

#### Diurnal changes in plasma substrates and tissue glycogen and triglyceride

**Fig. 3:**
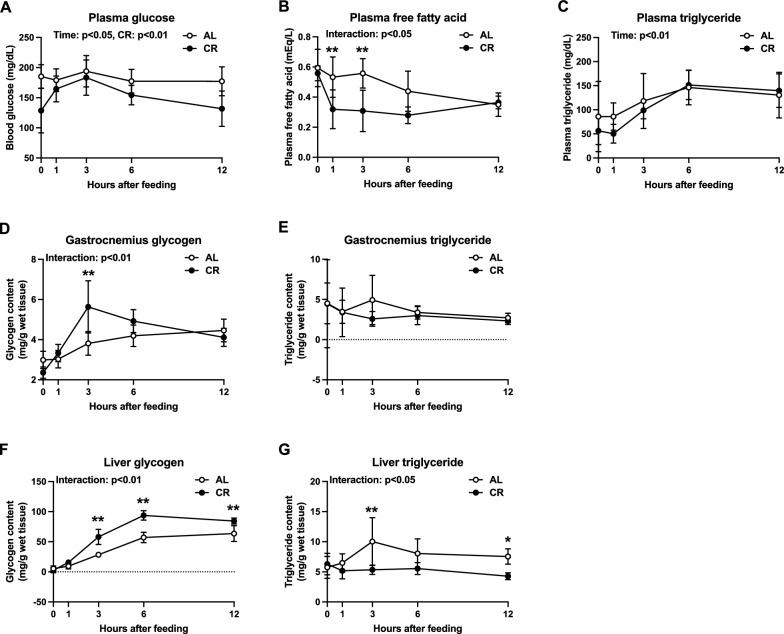
Plasma substrates and muscle and hepatic glycogen and triglyceride in Experiment 1. Diurnal changes in plasma glucose (**A**), free fatty acids (**B**), and triglyceride levels (**C**). Diurnal changes in the glycogen (**D**, **F**) and triglyceride (**E**, **G**) concentrations in the gastrocnemius muscle (**D**, **E**) and liver (**F**, **G**). The data are expressed as means ± the standard deviation of the mean (SD) (n = 5–6). Two-way analysis of variance (ANOVA) was performed to detect differences between two groups at the same time point. **p < 0.01: significant difference between the two groups within the same time point

Next, we evaluated the substrate levels in circulation after 7 days of CR. Main effects of time (p < 0.05) and CR (p < 0.01) on plasma glucose concentrations were observed (Fig. 3A). Plasma FFA levels at 1 and 3 h after feeding were significantly lower in the CR group than in the AL group (p < 0.01; Fig. 3B). Plasma triglyceride concentrations were not different between the two groups (Fig. 3C). Collectively, CR altered the plasma substrate concentration depending on the time of the day. We also determined the levels of glycogen and triglycerides, the main energy deposits in muscle and liver. In the gastrocnemius muscle, the glycogen content was significantly greater in the CR group than in the AL group 3 h after feeding (p < 0.01, Fig. 3D), whereas there were no significant differences in triglyceride concentration (Fig. 3E). In the liver, the glycogen content at 3, 6, and 12 h after feeding was significantly higher in the CR group compared to the AL group (p < 0.01, Fig. 3F). Additionally, hepatic triglyceride levels 3 h after feeding were significantly higher in the AL group versus the CR group (p < 0.01; Fig. 3G). These data indicate that CR induces time- and tissue-dependent changes in glycogen and triglyceride concentrations.

### Experiment 2

#### Respiratory gas exchange

**Fig. 4:**
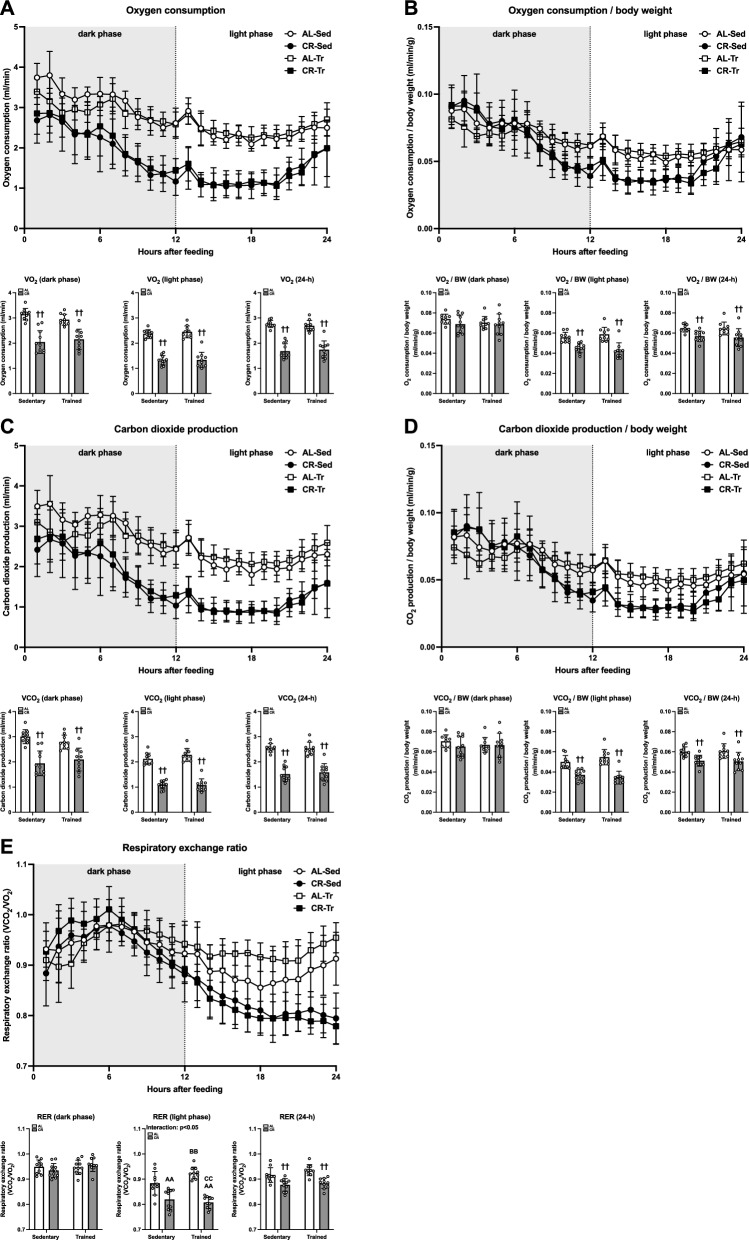
Oxygen consumption, carbon dioxide production, and respiratory exchange ratio in Experiment 2**.** A Oxygen consumption (**A**), carbon dioxide production **(C)**, respiratory exchange ratio (**E**) at rest. Oxygen consumption/body weight (**B**) and carbon dioxide production/body weight (**D**) at rest. The data are expressed as means ± the standard deviation of the mean (SD) (n = 9–11). A two-way analysis of variance (ANOVA) was performed to determine the interactions and main effects of ET and CR. ^††^p < 0.01: main effect of CR. ^#^p < 0.05: Main effect of ET. ^AA^p < 0.01: vs. AL-Sed. ^BB^p < 0.01: vs. CR-Sed. ^CC^p < 0.01: vs. AL-Tr

To clarify the impact of ET and CR on substrate metabolism, we evaluated respiratory gases during 24 h of rest. Figure 4A and B show VO_2_ and VO_2_/body weight, respectively. A negative main effect of CR was observed on average VO_2_, irrespective of the phase of the day (p < 0.01; Fig. 4A). Except during the dark phase, CR significantly decreased the VO_2_/body weight ratio (p < 0.01; Fig. 4B). Figure 4C and d show VCO_2_ and VCO_2_/body weight, respectively. Similar to VO_2_, CR significantly reduced the average VCO_2_ irrespective of the phase of the day (p < 0.01; Fig. 4C). Additionally, CR significantly decreased VO_2_/body weight during the all-day and light phases, but not during the dark phase (p < 0.01; Fig. 4D). RER results at rest are shown in Fig. 4E. A negative effect of CR was found at 24 h of average RER during the measurement (p < 0.01). Although the RER during the dark phase did not differ among the groups, the RER during the light was significantly greater in the AL-sedentary and AL-trained groups versus the CR-sedentary and CR-trained groups (p < 0.01).

#### Fuel oxidation and energy expenditure

**Fig. 5:**
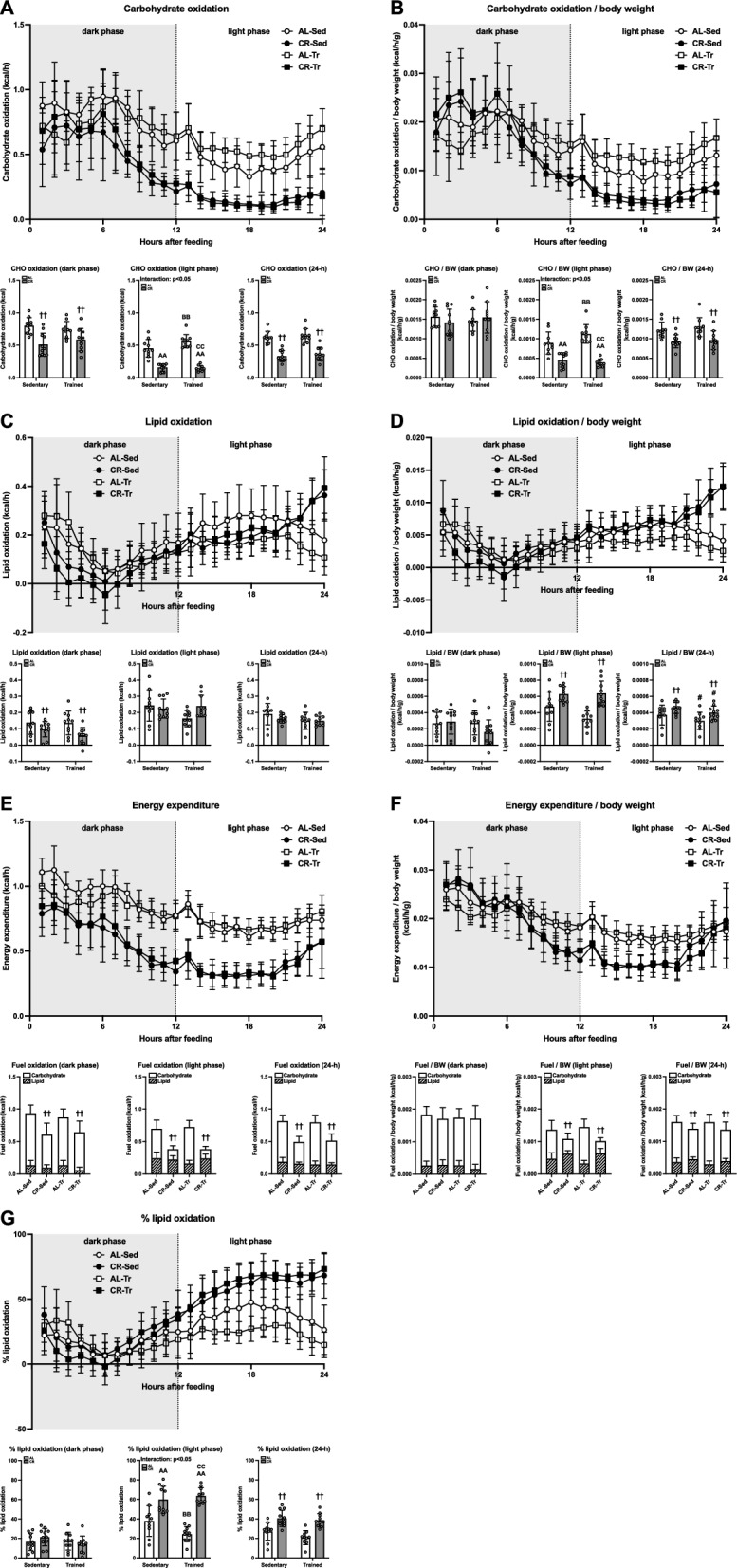
Fuel oxidation and energy expenditure in Experiment 2. Carbohydrate oxidation (**A**), lipid oxidation (**C**), energy expenditure (**E**), and percent lipid oxidation (**G**) at rest. Carbohydrate oxidation/body weight (**B**), lipid oxidation/body weight (**D**), and energy expenditure/body weight (**F**) at rest. The data are expressed as means ± the standard deviation of the mean (SD) (n = 9–11). A two-way analysis of variance (ANOVA) was performed to determine the interactions and main effects of ET and CR. ^††^p < 0.01: main effect of CR. ^AA^p < 0.01: vs. AL-Sed. ^BB^p < 0.01: vs. CR-Sed. ^CC^p < 0.01: vs. AL-Tr

The carbohydrate (CHO) oxidation and CHO oxidation/body weight data for 24 h are shown in Fig. 5A and B, respectively. There was a negative main effect of CR on the average CHO oxidation during 24 h and dark phases (p < 0.01, Fig. 5A and B). The average CHO oxidation and CHO oxidation/body weight ratio during the light phase were significantly greater in the AL-sedentary and AL-trained groups than those in the CR-sedentary and CR-trained groups (p < 0.01; Fig. 5A and B). Although the average CHO oxidation/body weight during the dark phase was similar between the groups, the average CHO oxidation/body weight over 24 h was significantly reduced by CR (p < 0.01; Fig. 5B). Lipid oxidation and lipid oxidation/body weight ratios after 24 h are presented in Fig. 5C and D, respectively. Although lipid oxidation during the dark phase was decreased by CR (p < 0.01, Fig. 5C), CR significantly increased lipid oxidation/body weight during the 24 h and light phases (p < 0.01, Fig. 5D). In contrast to CR, ET significantly decreased the 24-h lipid oxidation/body weight (p < 0.05, Fig. 5D). There were no significant effects on lipid oxidation during the 24 h and light phases, and on lipid oxidation/body weight during the dark phase (Fig. 5C and D). Figure 5E and F show the energy expenditure (EE) and EE/body weight, respectively. A negative main effect of CR on the average EE was observed, irrespective of the phase of the day (p < 0.01; Fig. 5E). Except during the dark phase, CR significantly decreased EE/body weight (p < 0.01, Fig. 5F). The percentage lipid oxidation (carbohydrate/lipid oxidation) was calculated as follows: Although the percentage of lipid oxidation did not differ during the dark phase, the percentage of lipid oxidation during the light phase was significantly greater in the AL-sedentary and AL-trained groups in comparison to the CR-sedentary and CR-trained groups (p < 0.01), resulting in a positive main effect of CR on the 24-h percent lipid oxidation (p < 0.01, Fig. 5G). Collectively, these results suggested that CR induces time-dependent alterations in substrate metabolism.

#### Body and tissue mass, and energy intake

**Fig. 6:**
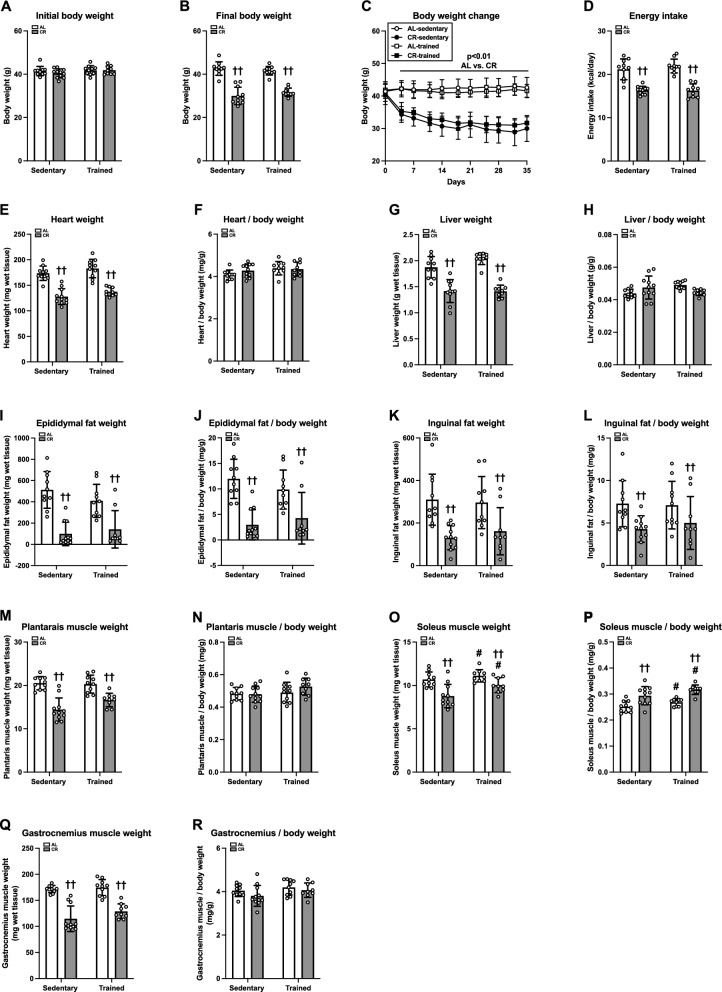
Body and tissue weights and energy intake in Experiment 2. Initial (**A**) and final (**B**) body weights. **c** Body weight changes during the experimental period (**C**). Daily energy intake during experimental period (**D**). Final weights of heart (**E**), liver (**G**), epididymal fat (**I**), inguinal fat (**K**), plantaris muscle (**M**), soleus muscle (**O**), and gastrocnemius muscle (**Q**). Relative weights of heart (**F**), liver (**H**), epididymal fat (**J**), inguinal fat (**L**), plantaris muscle (**N**), soleus muscle (**P**), and gastrocnemius muscle (**R**). The data are expressed as means ± the standard deviation of the mean (SD) (n = 9–11). A two-way analysis of variance (ANOVA) was performed to determine the interactions and main effects of ET and CR. Comparisons between two groups were made using the Student’s *t*-test. ^††^p < 0.01: main effect of CR. ^#^p < 0.05: Main effect of ET. ^AA^p < 0.01: vs. AL-Sed. ^BB^p < 0.01: vs. CR-Sed. ^CC^p < 0.01: vs. AL-Tr

Although the initial body weight did not differ among the groups (Fig. 6A), CR, but not ET, significantly reduced the body weight (p < 0.01, Fig. 6C), resulting in significantly lighter body weights in the CR group versus the AL group (p < 0.01, Fig. 6B). Energy expenditure during the experimental period was lower in the CR group compared to the AL group (p < 0.01; Fig. 6D). These observations suggest that CR, but not ET, effectively induced weight loss. Heart and liver weights decreased significantly after CR (p < 0.01; Fig. 6E and G). When normalized to the final body weight, the heart and liver weights did not differ between the groups (Fig. 6F and H). The epididymal and inguinal fat weights were also decreased by CR (p < 0.01; Fig. 6I and K). Even when normalized to body weight, these values remained lower in the CR group versus the AL group (p < 0.01; Fig. 6J and L). The weights of the plantaris, soleus, and gastrocnemius muscles decreased with CR (p < 0.01; Fig. 6M, O, and Q). Additionally, ET significantly increased the weight of the soleus muscle (p < 0.05, Fig. 6O). Although the plantaris and gastrocnemius muscles/body weights were similar between the groups (Fig. 6N and R), ET (p < 0.05) and CR (p < 0.01) additively increased the soleus muscle/body weight (Fig. 6P). These findings suggest that the CR-induced reduction in most, but not all, tissue weights is likely parallel to body size.

#### Plasma substrates and glycogen and triglyceride content in skeletal muscle and liver

**Fig. 7:**
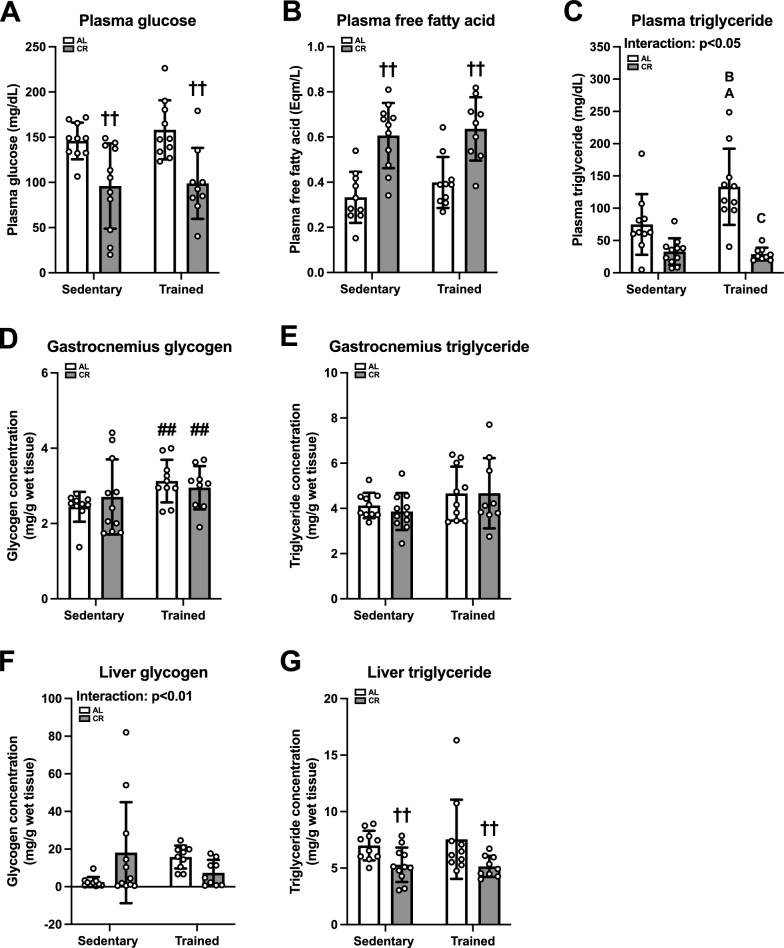
Plasma substrates and muscle and hepatic glycogen and triglyceride in Experiment 2. Plasma glucose (**A**), free fatty acids (**B**), and triglyceride (**C**) levels at rest. Glycogen (**D**, **F**) and triglyceride (**E**, **G**) concentrations in gastrocnemius muscle (**D**, **E**) and liver (**F**, **G**). The data are expressed as means ± the standard deviation of the mean (SD) (n = 9–11). A two-way analysis of variance (ANOVA) was performed to determine the interactions and main effects of ET and CR. ^††^p < 0.01: main effect of CR. ^##^p < 0.01: Main effect of ET. ^A^p < 0.05: vs. AL-Sed. ^B^p < 0.05: vs. CR-Sed. ^C^p < 0.05: vs. AL-Tr

Next, we evaluated circulating substrates during the experimental period. CR had a negative effect on plasma glucose concentration (p < 0.01; Fig. 7A). In contrast, a positive main effect of CR was detected on the plasma FFA concentration (p < 0.01, Fig. 7B). Plasma triglyceride levels were significantly elevated in the AL-trained group compared to other groups (p < 0.05; Fig. 7C). Thus, it is likely that CR exerts opposing effects on plasma glucose and FFA levels.

The glycogen and triglyceride concentrations in the gastrocnemius muscle and liver were assessed at the end of the experiment. ET had a positive main effect on gastrocnemius glycogen content (p < 0.01, Fig. 7D), whereas neither a significant interaction nor a main effect was detected for gastrocnemius triglycerides (Fig. 7E). Although we found a significant interaction between ET and CR in terms of hepatic glycogen levels (p < 0.01), we did not observe any significant differences between the groups (Fig. 7F). CR had a negative effect on hepatic triglyceride levels (p < 0.01; Fig. 7G). Overall, ET and CR likely changed the basal levels of substrates and energy deposits.

#### Glycolytic enzyme activity in skeletal muscle

**Fig. 8:**
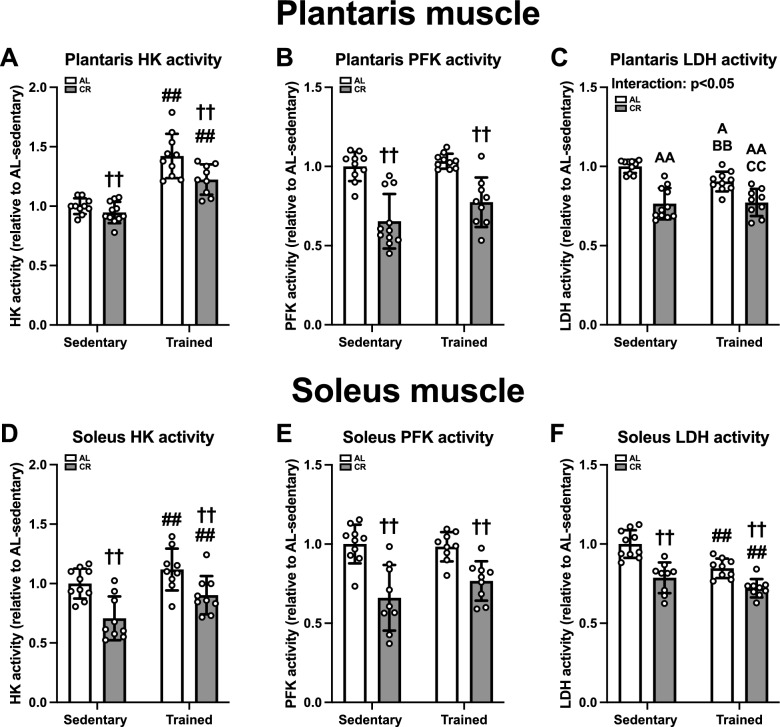
Glycolytic enzyme activities in the skeletal muscles of Experiment 2. Hexokinase (HK) (**A**, **D**), phosphofructokinase (PFK) (**B**, **E**), and lactate dehydrogenase (LDH) (**C**, **F**) activity in the plantaris (**A**–**C**) and soleus (**D**–**F**) muscles. Citrate synthase (CS) (**A**), cytochrome c oxidase (COX) (**B**), β-hydroxyacyl-CoA dehydrogenase (β-HAD) (**C**), and total carnitine palmitoyltransferase (CPT) (**D**) activities in the plantaris muscle. CS (**E**), COX (**F**), β-HAD (**G**), and total CPT (**H**) activities in the soleus muscle. The data are expressed as means ± the standard deviation of the mean (SD) (n = 9–11). A two-way analysis of variance (ANOVA) was performed to determine the interactions and main effects of ET and CR. ^††^p < 0.01: main effect of CR. ^##^p < 0.01: Main effect of ET. ^AA^p < 0.01, ^A^p < 0.05: vs. AL-Sed. ^BB^p < 0.01: vs. CR-Sed. ^CC^p < 0.01: vs. AL-Tr.

To characterize the metabolic outcomes after ET and CR, we first determined glycolytic enzyme activity. Although CR decreased the activity of HK and PFK in the plantaris and soleus muscles (p < 0.01, Fig. 8A, B, D, and E), HK activity in these muscles increased after ET (p < 0.01, Fig. 8A and B). Plantaris LDH activity was greater in the AL-sedentary group than in the other groups and in the AL-trained group than in the CR group (p < 0.01, Fig. 8C). Both ET and CR reduced the activity of LDH in the soleus muscle (p < 0.01; Fig. 8F). Overall, it is likely that CR reduces the skeletal muscle glycolytic capacity.

#### Mitochondrial enzyme activity and protein levels in skeletal muscle

**Fig. 9:**
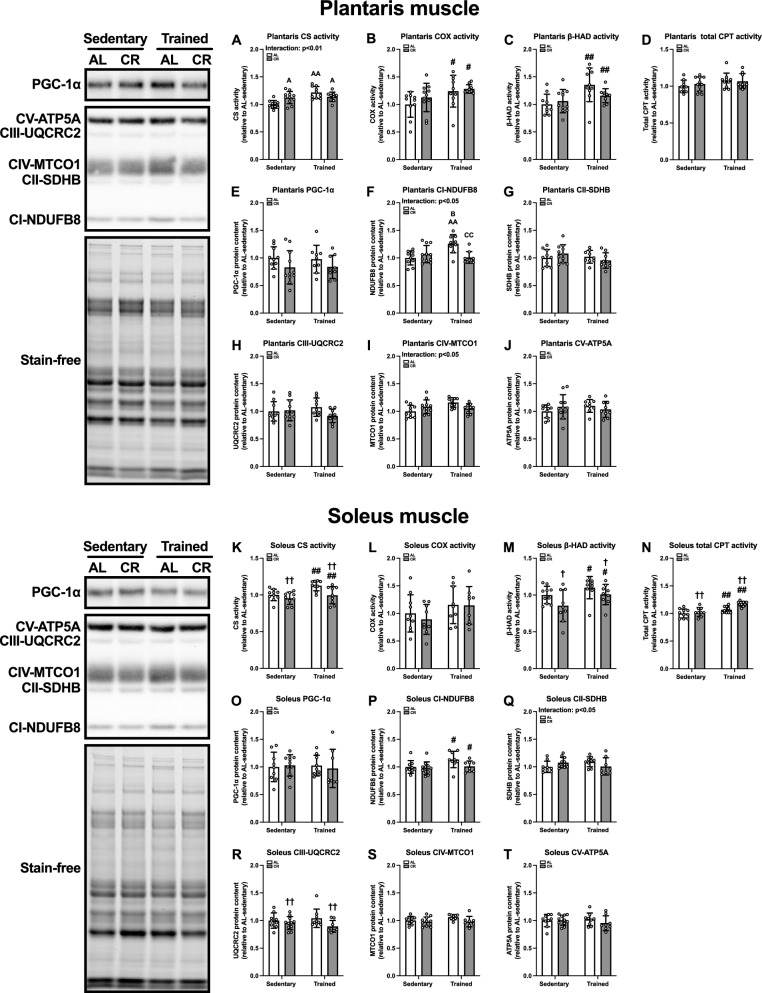
Mitochondria-associated enzyme activities and protein levels in the skeletal muscles of Experiment 2. Enzymatic activities of citrate synthase (CS) (**A**, **K**), cytochrome c oxidase (COX) (**B** and **I**), β-hydroxyacyl-CoA dehydrogenase (β-HAD) (**C**, **M**), and total carnitine palmitoyltransferase (CPT) (**D**, **N**) in the plantaris (**A**–**D**) and soleus (**K**–**N**) muscles. Protein levels of peroxisome proliferator-activated receptor γ coactivator 1-α (PGC-1α) (**E**, **O**), NADH:ubiquinone oxidoreductase subunit B8 (NDUFB8) (**F**, **P**), succinate dehydrogenase complex iron sulfur subunit B (SDHB) (**G**, **Q**), ubiquinol-cytochrome c reductase core protein 2 (UQCRC2) (**H**, **R**), mitochondrially encoded cytochrome c oxidase 1 (MTCO1) (**I**, **S**), and ATP synthase peripheral stalk subunit F6 (ATP5PF/ATP5A) (**J**, **T**) in the plantaris muscle. The data are expressed as means ± standard deviation of the mean (SD) (n = 9–11). A two-way analysis of variance (ANOVA) was performed to determine the interactions and main effects of ET and CR. ^††^p < 0.01, ^†^p < 0.05: main effect of CR. ^##^p < 0.01, ^#^p < 0.05: main effect of ET. ^AA^p < 0.01, ^A^p < 0.05: vs. AL-Sed. ^B^p < 0.05: vs. CR-Sed. ^CC^p < 0.01: vs. AL-Tr

We also determined enzyme activities and protein levels within the mitochondria, which are the major sites for bioenergetics. The CS activity in the plantaris muscle was elevated in the CR-sedentary (p < 0.05), AL-trained (p < 0.01), and CR-trained (p < 0.05) groups compared to the AL-sedentary group (Fig. 9A). Additionally, ET had a significant effect on plantaris COX (p < 0.05, Fig. 9B) and β-HAD (p < 0.01, Fig. 9C) activity. The protein abundance of PGC-1α, a key regulator of mitochondrial biogenesis, was not different among the groups (Fig. 9E). The protein level of NDUFB8 was significantly greater in the AL-trained group than that in the AL-sedentary (p < 0.01), CR-sedentary (p < 0.05), and CR-trained (p < 0.05) groups (Fig. 9F). There was no significant effect of ET or CR on the total CPT activity (Fig. 9D), SDHB (Fig. 9G), UQCRC2 (Fig. 9H), MTCO1 (Fig. 9I), or ATP5A (Fig. 9J) protein.

In the soleus muscle, the ET significantly enhanced the CS (p < 0.01, Fig. 9K) and β-HAD (p < 0.05, Fig. 9M) activities and NDUFB8 protein content (p < 0.05, Fig. 9P). However, the CR significantly decreased the CS (p < 0.01, Fig. 9K) and β-HAD (p < 0.05, Fig. 9M) activities, as well as the UQCRC2 protein content (p < 0.05, Fig. 9R). Both ET and CR significantly enhanced the total CPT activity (p < 0.01, Fig. 9N). There were no significant differences in the COX activity (Fig. 9L) or the PGC-1α (Fig. 9O), SDHB (Fig. 9Q), MTCO1 (Fig. 9S), or ATP5A (Fig. 9T) protein contents. These observations suggest a fiber type-specific mitochondrial adaptation to CR.

#### Substrate transporter proteins in skeletal muscle

**Fig. 10:**
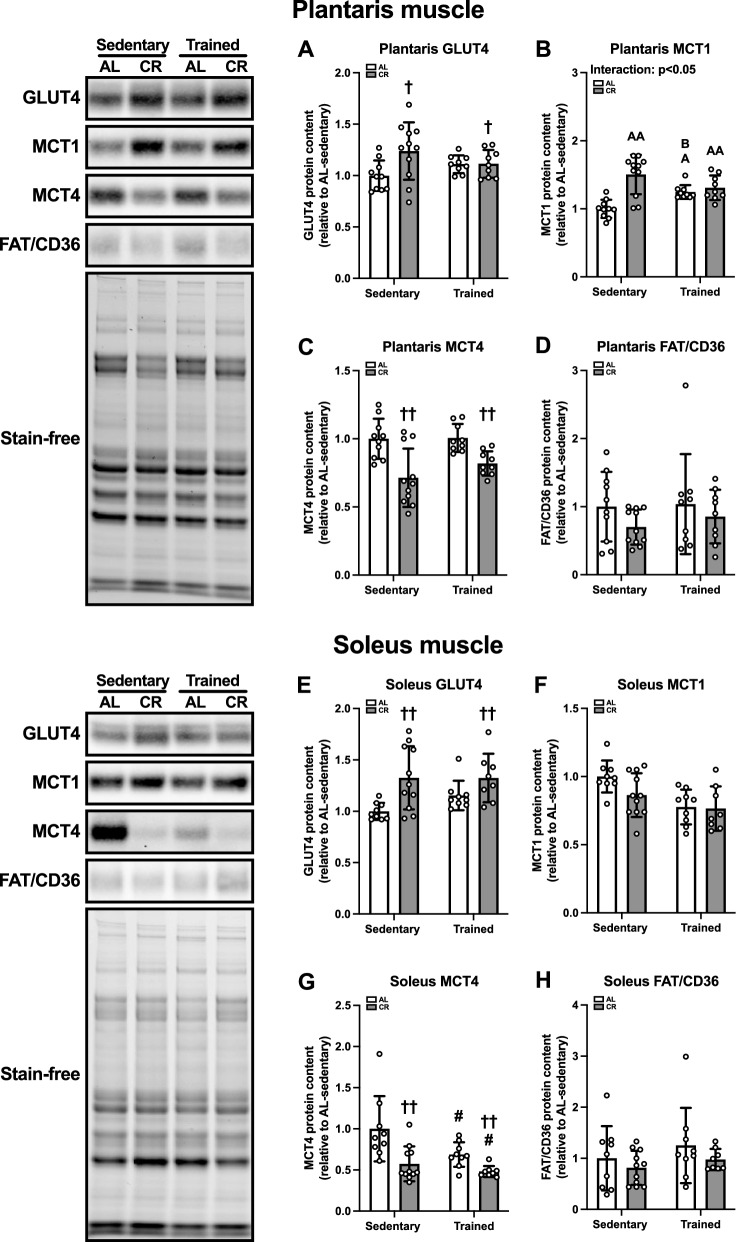
Substrate transport protein levels in the skeletal muscles of Experiment 2. Protein levels of glucose transporter 4 (GLUT4) (**A**, **E**), monocarboxylate transporter (MCT)-1 (**B**, **F**), MCT4 (**C**, **G**), and fatty acid translocase/cluster of differentiation 36 (FAT/CD36) (**D**, **H**) in the plantaris (**A**–**D**) and soleus (**E**–**H**) muscles. The data are expressed as means ± the standard deviation of the mean (SD) (n = 9–11). A two-way analysis of variance (ANOVA) was performed to determine the interactions and main effects of ET and CR. ^††^p < 0.01, ^†^p < 0.05: main effect of CR. ^#^p < 0.05: Main effect of ET

As substrate metabolism is expedited by its transport capacity, we evaluated substrate-specific transporters. CR significantly increased GLUT4 protein levels in the plantaris (p < 0.05, Fig. 10A) and soleus (p < 0.01, Fig. 10E) muscles. The plantaris MCT1 protein content was higher in the CR-sedentary (p < 0.01), AL-trained (p < 0.05), and CR-trained (p < 0.01) groups than in the AL-sedentary group and greater in the CR-sedentary group compared to the AL-trained group (p < 0.05) (Fig. 10B). In contrast, CR decreased MCT4 protein content in the plantaris (p < 0.01, Fig. 10C) and soleus (p < 0.01, Fig. 10G) muscles. ET also decreased MCT4 protein levels in the plantaris muscle (p < 0.05, Fig. 10G). There were no differences in the FAT/CD36 protein content of the plantaris (Fig. 10D) and soleus (Fig. 10H) muscles between the groups. Collectively, these results suggest that CR enhances the specific substrate uptake capacity of skeletal muscles.

#### Gluconeogenic, lipogenic, and mitochondrial proteins in liver

**Fig. 11:**
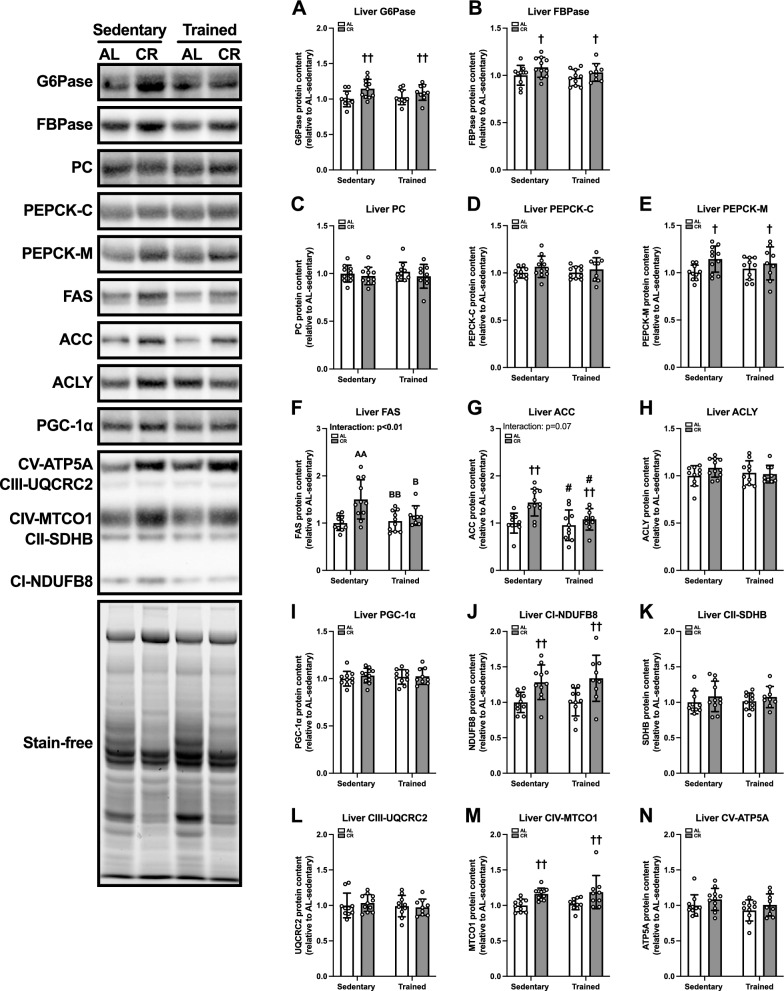
Protein levels related to gluconeogenic, fatty acid synthesis, and mitochondria in the liver of Experiment 2. Protein levels of glucose 6-phosphatase (G6Pase) (**A**), fructose 1,6-bisphosphatase (FBPase) (**B**), pyruvate carboxylase (PC) (**C**), cytosolic phosphoenolpyruvate carboxykinase (PEPCK-C) (**D**), mitochondrial phosphoenolpyruvate carboxykinase (PEPCK-M) (**E**), fatty acid synthase (FAS) (**F**), acetyl-CoA carboxylase (ACC) (**G**), ATP citrate synthase (ACLY) (**H**), peroxisome proliferator-activated receptor γ coactivator 1-α (PGC-1α) (**I**), NADH:ubiquinone oxidoreductase subunit B8 (NDUFB8) (**J**), succinate dehydrogenase complex iron sulfur subunit B (SDHB) (**K**), ubiquinol-cytochrome c reductase core protein 2 (UQCRC2) (**L**), mitochondrially encoded cytochrome c oxidase 1 (MTCO1) (**M**), and ATP synthase peripheral stalk subunit F6 (ATP5PF/ATP5A) (**N**) in the liver. The data are expressed as means ± the standard deviation of the mean (SD) (n = 9–11). A two-way analysis of variance (ANOVA) was performed to determine the interactions and main effects of ET and CR. ^††^p < 0.01, ^†^p < 0.05: main effect of CR. ^#^p < 0.05: Main effect of ET. ^AA^p < 0.01: vs. AL-Sed. ^BB^p < 0.01, ^B^p < 0.05: vs. CR-Sed

Given that the liver is central to substrate supply in response to nutritional status, we measured the proteins mediating the synthesis of glucose and fatty acids in the liver. Regarding gluconeogenesis, CR significantly increased the protein abundance of G6Pase (p < 0.01, Fig. 11A), FBPase (p < 0.05, Fig. 11B), and PEPCK-M (p < 0.05, Fig. 11E). There was no significant effect on the PC (Fig. 11C) or PEPCK-C (Fig. 11D) protein content. Concerning fatty acid synthesis, the FAS protein content in the CR-sedentary group was significantly greater than that in the AL-sedentary (p < 0.01), AL-trained (p < 0.01), and CR-trained (p < 0.05) groups (Fig. 11F). Additionally, ACC protein level was significantly enhanced by CR (p < 0.01) but reduced by ET (p < 0.05) (Fig. 11G). There was no significant interaction or effect on ACLY protein content (Fig. 11H). Collectively, these results suggest that CR enhances the gluconeogenic and lipogenic capacities of the liver.

According to previous studies, the hepatic mitochondrial content increases in response to CR. We therefore evaluated the protein levels of PGC-1α and mitochondrial respiratory chain components. CR increased the protein levels of NDUFB8 (p < 0.01; Fig. 11J) and MTCO1 (p < 0.01; Fig. 11M) in the liver. No significant effects of the ET and CR were noted in the PGC-1α (Fig. 11I), SDHB (Fig. 11K), UQCRC2 (Fig. 11L), or ATP5A (Fig. 11N) protein contents. Collectively, these results suggest that CR may increase mitochondrial volume density in the liver.

## Discussion

### Body and tissue weight changes

In the present study, we found that CR, but not ET, significantly decreased body and adipose weights. Similarly, a previous human study demonstrated that CR, but not resistance training, significantly decreased body and fat weight [28]. However, another human study showed that CR and ET reduced body and fat weights to similar extents when they induced the same negative energy balance [29]. Collectively, it is likely that the degree of weight and fat loss following CR and exercise training depends on the amount of energy deficit but not on the type of intervention.

### Substrate metabolism at rest

The experiments presented here replicate a previous observation that CR administered by daily feeding results in a unique pattern of fuel selection in mice, exhibiting an initial increase in RER followed by a gradual decrease in RER [15, 16]. The increase in RER after the onset of feeding suggests that animals subjected to CR become carbohydrate metabolism-dominant and fatty acid synthesis from carbohydrates occurs. Several hours later, animals with CR appeared to shift from carbohydrate to fat metabolism, resulting in a gradual decrease in the RER. In Experiment 1, on the first day of the CR, the animals did not consume the entire daily food allotment until 12 h after feeding. After 7 days of CR, the animals consumed the entire daily food allotment within 3 h of feeding, suggesting that CR reestablished feeding patterns within a week. After consuming food allotments daily and spending on carbohydrate energy deposits, animals subjected to CR begin to rely on lipids as an energy source. Thus, fuel selection during CR is likely associated with feeding patterns.

It is well known that ET enhances lipid metabolism during the same absolute intensity exercise, as we have previously demonstrated [22]. In the current study, no significant effect of ET on RER or the percentage of lipid oxidation was observed, suggesting that ET does not significantly alter substrate metabolism. This was probably because we performed the respiratory gas analysis when the animals were at rest. Future studies should investigate whether the ET and CR alter fuel selection during exercise.

### Glycolytic capacity in skeletal muscle

In glycolysis, HK regulates glucose phosphorylation and, therefore, glucose uptake in the skeletal muscle, whereas PFK controls the overall glycolytic flux. Additionally, MCT4 primarily facilitates lactate efflux from the skeletal muscles, which prevents suppression of glycolysis. In the present study, CR induced decreases in HK, PFK, and LDH activities, as well as MCT4 protein content in the plantaris and soleus muscles, suggesting a decline in glycolytic capacity. Although ET enhances HK activity in the plantaris and soleus muscles, extracellular glucose provides little total glycolysis during intense exercise [30]. Collectively, ET is unlikely to offset the CR-induced loss of glycolytic capacity in the skeletal muscle.

In this study, animals in the CR training group performed ET 1 h after feeding. In Experiment 1, the muscle and liver glycogen contents were comparable between the AL and CR groups 1 h after feeding. This observation suggests that the animals in the CR training group did not undergo ET due to low carbohydrate availability. In Experiment 2, the plasma glucose levels in animals subjected to CR decreased 24 h after feeding, suggesting that carbohydrate availability decreased during a certain period of the day. Taken together, our results indicate that carbohydrate availability throughout the day, rather than during exercise, is important for maintaining the glycolytic capacity of skeletal muscles.

### Mitochondrial enzyme activity and protein levels in skeletal muscle

Several studies have reported that CR increases the mitochondrial volume density in skeletal muscles [7, 8, 31]. In this study, CR alone enhanced the activity of CS in the plantaris muscle. Although CS activity is a classic marker of skeletal muscle mitochondrial volume density [32], it is questionable whether the current observation of enhanced CS activity after CR alone indicates an increase in mitochondrial volume density in the plantaris muscle, as CR does not alter other mitochondrial enzyme activities and proteins. In the soleus muscle, CR declined the CS and β-HAD activities, as well as the UQCRC2 protein content, although the total CPT activity increased after the CR. The dissociation of mitochondrial content markers between the plantaris and soleus muscles could be attributed to the differences in the recruitment patterns of the fiber types. In compliance with the size principle, slow-twitch fibers are recruited at low-intensity workloads, whereas fast-twitch fibers become active at higher intensities [33, 34]. While the plantaris muscle consists of more than 90% fast-twitch fibers, the soleus muscle is composed of only approximately 50% fast-twitch fibers [35]. These data suggest that slow-twitch fibers are mainly used in daily physical activity, and that the soleus muscle is more likely to be sensitive to a decline in daily physical activity. The current observation of lower oxygen consumption during CR may indicate a decline in voluntary physical activity. Collectively, the loss of mitochondrial enzyme activity and protein content in the soleus muscle may be partly attributed to the CR-induced decline in physical activity.

Mitochondrial volume density in a tissue depends on the balance between the synthesis and degradation of the tissue and mitochondria. In the current report, no changes were observed in the protein content of PGC-1α, a major regulator of mitochondrial biogenesis [36], in the plantaris or soleus muscle. Other investigators have shown that the PGC-1α expression and mitochondrial fractional synthesis did not change during CR [37, 38]. These data suggest that alterations in mitochondrial enzyme activity and protein levels are unlikely to be explained by biogenesis. In terms of tissue weight, CR reduced both the plantaris and soleus muscle weights. However, the extent of the muscle weight loss was larger in the plantaris muscle (− 24.4%) rather than the soleus muscle (− 14.2%). Taken together, the dissociation of mitochondrial enzyme activity and protein content between muscle types may result, in part, from the different degrees of muscle atrophy.

Another explanation for skeletal muscle-dependent changes in enzyme activity and protein levels is the workload imposed on the muscles. Currently, CR does not significantly alter plantaris muscle weight relative to body weight but significantly increases soleus muscle weight relative to body weight. These observations suggest that the workload imposed on the soleus muscle decreased after CR, whereas that imposed on the plantaris muscle remained unchanged. Decreased workload in the soleus muscle may have contributed to the decline in enzyme activity and protein levels of mitochondria after CR.

It is well accepted that ET enhances mitochondrial oxidative capacity in skeletal muscle [12]. Accordingly, we observed significant increases in mitochondrial enzyme activities and protein levels following ET. These increases appeared to offset the CR-induced decline in mitochondrial indices in the soleus muscle. However, the combination of CR and ET did not additively or synergistically enhance mitochondrial enzyme activity (except for total CPT activity in the soleus muscle) or protein levels in muscles, suggesting that CR does not augment the effects of ET on muscle oxidative capacity. Notably, previous studies have reported improved mitochondrial respiratory function without changes in mitochondrial content markers in mouse skeletal muscle following weight loss [39, 40]. Additionally, ET enhances skeletal muscle mitochondrial respiratory function [41]. Further studies are required to clarify the interaction between ET and CR in the mitochondrial respiratory function of the skeletal muscles.

### Substrate transport proteins in skeletal muscle

Several studies have demonstrated that CR improves insulin-stimulated glucose uptake by enhancing several steps in the insulin signaling pathway, resulting in increased GLUT4 translocation in skeletal muscle [42–44]. Several studies have demonstrated that CR increases protein levels of GLUT4 in skeletal muscles [42, 45, 46]. The current observation that CR enhances GLUT4 protein content supports the results of previous studies. Given that insulin-stimulated muscle glucose uptake is proportional to GLUT4 protein content [47], CR-induced increases in muscle GLUT4 protein content are expected to improve glucose homeostasis.

Similar to GLUT4, the MCT1 protein levels in the plantaris muscle increased after CR. As MCT1 mainly facilitates lactate uptake, this observation may indicate an enhanced lactate uptake capacity in the plantaris muscle. We and others have reported that GLUT4 and MCT1 protein levels increase in patients with McArdle disease, who are unable to use muscle glycogen because of a lack of phosphorylases [48, 49]. Taken together, these results indicate that the loss of intramuscular carbohydrate availability leads to enhanced glucose and lactate uptake capacity within skeletal muscle. The lack of change in MCT1 protein content in the soleus muscle may be because the soleus muscle is less likely to consume glycogen because of the abundance of mitochondria compared to the plantaris muscle.

Fatty acid uptake is facilitated FAT/CD36. In the present study, no difference in FAT/CD36 protein content was observed despite increased fat metabolism during CR. Inconsistencies between lipid metabolism and FAT/CD36 protein levels have been reported in previous studies [50], including ours [22, 51]. Although FAT/CD36 is considered to facilitate fatty acid uptake and, therefore, fat metabolism [52], FAT/CD36 is not likely to be a rate-regulating factor for lipid metabolism under the current experimental conditions in which the animals were at rest. Instead, lipid metabolism is likely to be regulated by interactions with carbohydrate metabolism.

### Impacts of CR on ET-induced skeletal muscle adaptations

In the present study, CR-induced energy insufficiency partially reduced skeletal muscle adaptation to ET. For example, ET and CR alone, but not in combination, enhanced CS activity and NDUFB8 and MCT1 proteins in the plantaris muscle. A previous report showed that ET and high-fat diet feeding, which result in excess energy intake, additively enhanced enzyme activity of rat skeletal muscle mitochondria [53]. Another research showed that exercise training and consumption of a high-fat diet additively enhanced lipid oxidation during acute bouts of exercise [54]. These effects of ET under HFD-fed conditions contrast with the present observation of no additive effects of ET and CR, suggesting that adequate energy intake is necessary to derive the beneficial effects of ET.

### Hepatic adaptations

The liver has a crucial role in substrate conversion in response to the nutritional status. Previous work has reported that CR increases the mRNA expression of G6Pase, PEPCK, and PEPCK in the mouse liver [55]. Consistent with these findings, we observed that CR increased the protein levels of G6Pase, FBPase, and PEPCK-M. These data suggested that CR enhanced the enzymatic capacity for gluconeogenesis in the liver. Several hours after consuming the entire daily food allotment, the dietary sources of carbon for maintaining blood glucose levels are exhausted. Under these circumstances, the liver is capable of synthesizing glucose from other carbon skeletons such as amino acids, lactate, and glycerol. Thus, an increase in gluconeogenesis likely prevents hypoglycemia.

Previous reports have shown that the mRNA levels of FAS and ACC decrease during CR [56–59], suggesting that CR decreases fatty acid synthesis. However, another study reported that immediately after food was provided, the mRNA levels of FAS and ACC increased, leading to higher values than those in AL controls [15]. In the current study, we determined the FAS and ACC protein contents just before food supply and found an increase in these proteins, suggesting that CR enhances the capacity for hepatic fatty acid synthesis and that lipogenic genes and proteins do not change proportionately. The increase in fatty acid synthesis appears to be reasonable, given that not all consumed carbohydrates can be stored as glycogen simultaneously, but they are synthesized into fatty acids and incorporated into triglycerides, which are used when carbohydrate availability decreases.

According to previous studies, CR increases the mitochondrial volume density in the liver by promoting mitochondrial biogenesis [6, 7]. Here, we found that CR increased the expression of mitochondrial proteins NDUFB8 and MTCO1 in the liver. However, the observed changes in mitochondrial protein contents are unlikely to result from increased mitochondrial biogenesis because the PGC-1α protein content did change. In support of this view, a previous study reported no significant changes in PGC-1α protein content or mitochondrial fractional synthesis in mouse liver during CR [37]. As described above, the mitochondrial volume density in a tissue is determined by mitochondrial synthesis and breakdown. In the present study, CR significantly decreased liver weight. Thus, a rapid drop in hepatic mass relative to mitochondrial degradation may have increased mitochondrial protein content in the liver.

### Limitations and future perspectives

We should note the limitation in that we did not evaluate insulin levels. Secretion of insulin in response to nutritional intake. In this study, on day 7 of the CR, the animals consumed the entire daily food allotment within 3 h of feeding, suggesting that the insulin secretion pattern differed between the AL and CR groups. Previous studies have reported that STZ-induced insulin deficiency reduces glycolytic and mitochondrial enzyme activity in rodent skeletal muscle [60–62]. Therefore, it is possible that the action of insulin on skeletal muscles influences the metabolic function and substrate selection. Future studies are needed to clarify the pattern of insulin secretion during CR and its relation to metabolic adaptation in the skeletal muscle and liver.

## Conclusions

In this study, we evaluated the combined effects of ET and CR on substrate metabolism. In addition to decreasing carbohydrate consumption at rest, CR significantly decreased the activity of glycolytic enzymes (HK, PFK, and LDH) in the plantaris and soleus muscles. Although ET significantly enhanced mitochondrial enzyme activity (CS, COX, and β-HAD) and protein (NDUFB8) levels in the plantaris and soleus muscles, most of the mitochondrial enzyme activities and protein levels (except for CS activity in the plantaris muscle and total CPT activity in the soleus muscle) did not change or even decreased following CR. Additionally, CR significantly increased GLUT4 protein in the plantaris and soleus muscles, and MCT1 protein in the plantaris muscle. In the liver, CR increased gluconeogenic (G6Pase, FBPase, and PEPCK-M) and lipogenic (FAS and ACC) protein levels. These observations suggest that CR enhances the capacity for glucose and fatty acid synthesis in the liver, as well as the glucose and lactate uptake capacity in skeletal muscle, while reducing carbohydrate consumption in skeletal muscle. This suggests that ET during CR does not have a significant impact on substrate metabolism at rest. Future studies are needed to clarify whether the combination of ET and CR effectively alters mitochondrial function and substrate metabolism during exercise.

## Data Availability

The datasets used and/or analyzed in the current study are available from the corresponding author upon reasonable request.

## Abbreviations

β-HAD: β-Hydroxyacyl-CoA dehydrogenase
ACC: Acetyl-CoA carboxylase
ACLY: ATP citrate lyase
ANOVA: Analysis of variance
ATP5PF/ATP5A: ATP synthase peripheral stalk subunit F6
COX: Cytochrome c oxidase
CPT: Carnitine palmitoyltransferase
CR: Calorie restriction
CS: Citrate synthase
ET: Endurance training
FAS: Fatty acid synthase
FAT/CD36: Fatty acid translocase/cluster of differentiation 36
FBPase: Fructose 1,6-bisphosphatase
G6Pase: Glucose 6-phosphatase
GLUT: Glucose transporter
HIF-1α: Hypoxia-inducible factor 1-α
HK: Hexokinase
ICR: Institute of Cancer Research
LDH: Lactate dehydrogenase
MCT: Monocarboxylate transporter
MTCO1: Mitochondrially encoded cytochrome c oxidase 1
NDUFB8, NADH: Ubiquinone oxidoreductase subunit B8
PC: Pyruvate carboxylase
PEPCK: Phosphoenolpyruvate carboxykinase
PFK: Phosphofructokinase
PGC-1α: Peroxisome proliferator-activated receptor γ coactivator 1-α
RER: Respiratory exchange ratio
SDHB: Succinate dehydrogenase complex iron sulfur subunit B
UQCRC2: Ubiquinol-cytochrome c reductase core protein 2
